# Cavitation-Effect-Based Treatments and Extractions for Superior Fruit and Milk Valorisation

**DOI:** 10.3390/molecules28124677

**Published:** 2023-06-09

**Authors:** Madalina-Petruta Bucur, Maria-Cristina Radulescu, Gabriel Lucian Radu, Bogdan Bucur

**Affiliations:** National Institute of Research and Development for Biological Sciences, Centre of Bioanalysis, 296 Splaiul Independentei, 060031 Bucharest, Romania

**Keywords:** extraction, cavitation, ultrasounds, fruit, milk

## Abstract

Ultrasound generates cavities in liquids with high-energy behaviour due to large pressure variations, leading to (bio)chemical effects and material modification. Numerous cavity-based treatments in food processes have been reported, but the transition from research to industrial applications is hampered by specific engineering factors, such as the combination of several ultrasound sources, more powerful wave generators or tank geometry. The challenges and development of cavity-based treatments developed for the food industry are reviewed with examples limited to two representative raw materials (fruit and milk) with significantly different properties. Both active compound extraction and food processing techniques based on ultrasound are taken into consideration.

## 1. Introduction

**Figure 1:**
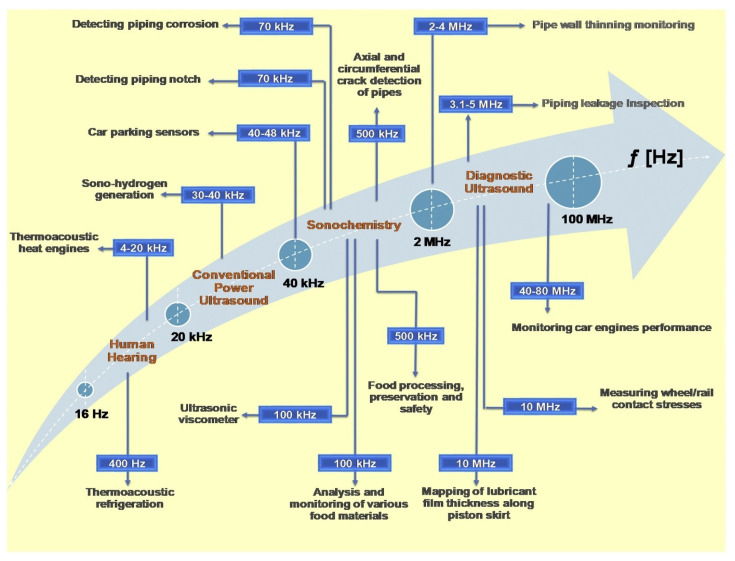
The frequency domain of sound and several corresponding applications. Reproduced from [5] with permission.

Ultrasound with high frequencies has numerous practical applications, some well known to the general public (ultrasonic tooth cleaning or medical imaging of internal organs), but various research and industrial fields take advantage of the high-energy cavities generated by ultrasound (Figure 1). The processes of the food industry are highly diverse and benefit from the inclusion of ultrasound treatments with various aims, such as pasteurisation with minimal impacts on flavour [1], enhanced seed oil extraction [2], the extraction of high-value-added compounds from fruit by-products [3] or pathogen inactivation [4]. Obviously, such diverse applications imply different working set-ups and operational conditions, and the highly diverse properties of the raw materials only add to the complexity of the field. The vibrant research activities aiming to develop novel food processing techniques with superior performance are only slowly being translated into real industrial applications due to up-scaling difficulties, raw material variability and legislative demands. This review aims to provide a bridge from theoretical aspects of the cavitation and phenomena that occur in materials during the process of acoustic cavitation to available ultrasound sources/extractors and parameters that influence extraction processes, reaching finally the processing and extraction of two relevant raw materials: milk and fruit. Only with a deep understanding of the overall process chain and theoretical and industrial constraints is it possible to successfully valorise the output of researchers.

## 2. Theoretical Aspects of Cavitation Phenomena

Cavitation phenomena represent the generation of cavities with intense dynamic behaviour when a liquid suffers from a large pressure variation and is manifested by a high temperature and pressure, microjets, shock waves and light emission, which also induce (bio)chemical effects. Processes based on the cavitation phenomenon have been widely used in many fields, such as sonochemistry, environmental engineering, biomedical engineering, materials science, food processing, object cleaning, extraction, cutting solids, etc. [6]. In order to generate cavitation phenomena, it is necessary to use an energy source (mainly ultrasound) that induces disturbances in the working liquid. Sound is the propagation of mechanical waves in different gaseous, liquid and solid media. Humans are able to discern only a range of sound frequencies, sounds with lower frequencies being called infrasound and those with a higher frequency being called ultrasound [7], and numerous practical applications are developed at different frequencies [5] (Figure 1).

The generation of ultrasound is based on the piezoelectric effect: electrical energy is transformed by piezoelectric materials into mechanical vibrations with a controlled frequency. The piezoelectric effect is in fact bidirectional. If mechanical pressure is applied to two opposite faces of a piezoelectric material, an equal and opposite electric charge develops on the other faces, resulting in a potential difference. Conversely, if a potential difference is applied to two opposite faces of the piezoelectric material, then mechanical movement of the other faces is produced depending on the direction of the applied potential difference. The piezoelectric effect has been observed in many materials, including quartz (used to construct watches or devices used in analytical chemistry), tourmaline and Roche salt [8].

**Figure 2:**
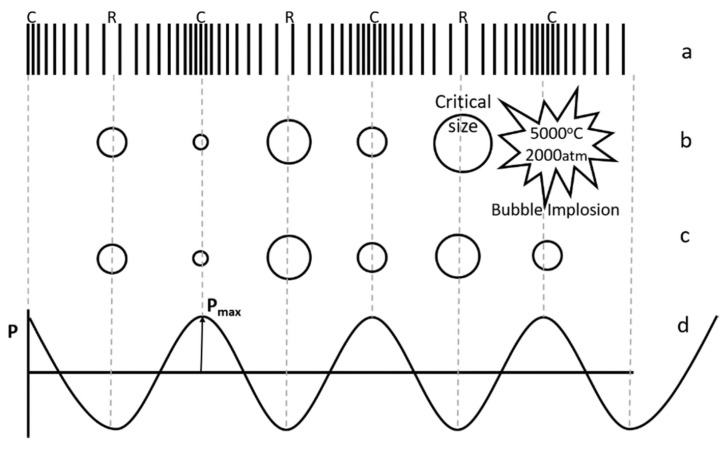
Ultrasonic wave propagation: (**a**) compression and rarefaction, (**b**) stable ultrasonic cavitation, (**c**) transient ultrasonic cavitation, (**d**) pressure variation during compression and rarefaction [10]. C is compression, R is rarefaction, and P is pressure.

Similarly to all sound waves, ultrasonic waves are also transmitted through transverse vibrations, compressing and stretching the molecular distance of the medium through which they pass. During the negative pressure cycle, the liquid is separated in places that contain a gaseous impurity, known as a “weak point” in the liquid. When the negative pressure is high enough, the distance between the liquid molecules exceeds the maximum molecular distance necessary to keep the liquid in contact, and then the liquid breaks down, creating numerous voids. These gaps are called “cavities”, whose size can be roughly estimated by the equation F × R = 3, where F is the frequency in MHz and R is the radius in microns [8]. The evolution of the formation, growth and collapse of the bubbles [9] is presented in Figure 2.

The effects in the propagation environment are different depending on the power of the ultrasound. Low-power ultrasound does not mechanically or chemically change the material and offers good sensitivity (echolocation, ultrasound), but, at high power, it induces physical and chemical changes in the medium through which it passes via the cavitation phenomenon. The cavity is produced from the collapse of the bubbles inside the medium, which leads to high pressure and temperatures in localised areas [7].

The mathematical modelling of cavitation phenomena allows the estimation of numerous parameters, such as the spatial variation of chemical species and energy in the bubbles and in the surrounding liquid, and the chemical reactions produced by the dissociation of molecules at different working temperatures. Thus, the amount of reactive free radicals produced during the collapse of the bubble at the saturation temperature is much higher compared to the same wave at room temperature, due to the higher concentration of vapour molecules in the bubble. The evolution of the bubble radius, the variations in different types of energy during the cavitation and the evolution over time of the reactive species created as a result of the energy conversion [11] must be taken into account when developing sample processing methods, depending on the purpose of the application—extraction, pasteurisation, homogenisation, etc.—to avoid the distortion of the products to be extracted or obtained.

**Figure 3:**
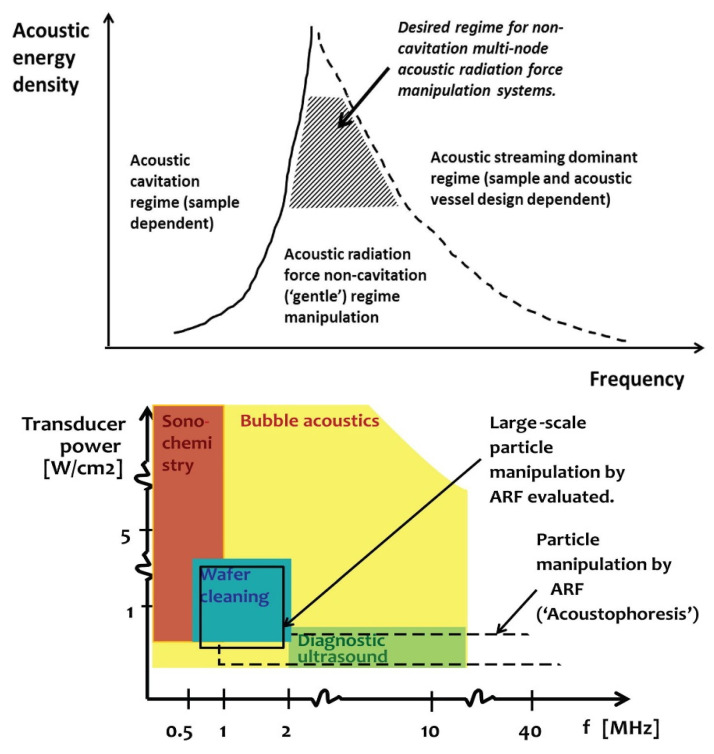
The operational parameter regions optimised for different ultrasound applications. Reprinted from [12] with permission.

The optimal operational parameters of ultrasound treatments follow different goals: the power-dependent cavitation threshold and, respectively, the production or not of free radicals. High acoustic power in large-scale ultrasonic separation is useful when working with high flow rates, but it must be below the level at which particle alignment is disturbed. The optimal power level depends on the sonicator parameters and the reactor geometry. In addition, there are specific applications that use ultrasound in non-cavitation mode. The operational parameters allow the handling of the samples with several regimes: operation in conditions without cavitation (“mild”) and, respectively, with cavitation (Figure 3). For use in a regime without cavitation, low frequencies must be avoided because the cavitation activity increases with the increase in the pressure amplitude and with the increase in the frequency until the peak values are reached. Biomedical applications are cavity-free, but this regime also applies in other situations when mixing or separation is desired in order to avoid chemical reactions evidenced by chemiluminescence [12].

## 3. Phenomena That Occur in Materials during the Process of Acoustic Cavitation

The sonochemical effects of ultrasound in a liquid are attributed to acoustic cavitation phenomena [13]: the bubble formation, growth and implosion that occur during the propagation of an ultrasound wave in a liquid medium [14]. The constituent molecules of the liquid medium are held together by attractive forces, but the transverse propagation of an ultrasound wave through an elastic medium induces compression and rarefaction phases, which result in the longitudinal displacement of these constituent molecules. The molecules that form the liquid phase are temporarily displaced from their initial positions and, during the compression cycles, collide with the surrounding molecules. During the rarefaction phase, a negative pressure will be exerted that depends on the nature and purity of the liquid [10]. At a sufficiently high intensity, during a rarefaction phase, the forces of attraction between molecules can be overcome, generating a cavity in the liquid (bubbles) [10]. These cavitation bubbles are able to grow by coalescence and/or diffusion [15] because the vapour or dissolved gas will not be completely expelled during the compression cycle. Cavity bubbles are of two types: stable and transient. Transient cavitation bubbles exist for one or, at most, several acoustic cycles, during which they expand very rapidly to at least double their initial size, before violently collapsing into smaller bubbles [10]. When the size of these bubbles reaches a critical value, they collapse during a compression cycle and a transient hot spot is created [12]. The collapse of the cavitation bubbles generates extreme local conditions: temperatures determined up to approximately 5000 K and pressures estimated at around 50–1000 atm [13]. These critical points are capable of dramatically accelerating the substance’s chemical reactivity or extraction [12]. If the acoustic cavity bubbles collapse on the surface of a solid material, a series of additional physical effects occur [13]. The collapse of the bubble generates jets of liquid at high speed, which creates damage to the solid surface through shock waves. These effects can lead to the fragmentation of friable materials and localised erosion. In the case of a solid–liquid mixture, the acoustic cavity and shock waves induce intense macro-turbulence, micro-mixing and collisions between particles [16]. All these phenomena lead to increased reactivity and an increase in the mass transfer of substances of interest (extraction).

**Figure 4:**
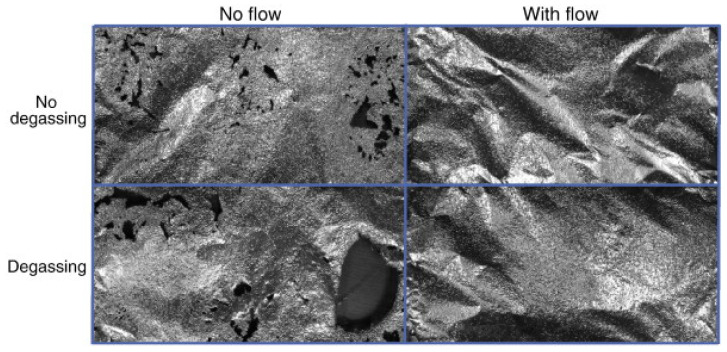
The damage to aluminium foil treated for 30 s in ultrasonic washing channel in various working conditions. Reprinted from [18] with permission.

The cavitation phenomena induce numerous macro- and microscopic effects in the structure of the food material from which the extraction is performed. Fragmentation of the samples takes place during the application of ultrasound in a liquid that also contains a vegetable raw material. Fragmentation can occur due to collisions between particles and from shock waves created from the collapse of cavitation bubbles. A direct consequence of the reduction in particle size through the action of ultrasound is the increase in the contact surface between the solid and liquid, resulting in increased mass transfer and an improved extraction yield. In comparison with the conventional extraction process by maceration, which does not lead to a reduction in the particle size, much faster extraction kinetics were obtained [16]. Moreover, other specific phenomena can appear in the fragmentation process under the influence of ultrasound, besides the fragmentation of the sample particles. For example, a specific surface “erosion” process was observed when treating boldo leaves with a 20 kHz ultrasonic probe: a localised effect on the trichomes (perisoria) that were specifically removed. The plant material was not fragmented in this situation, but the removal of the trichomes led to a specific degradation that had the effect of increased accessibility of the solvent, leading to an increase in the extraction speed and an improvement in the yield from 20% for maceration to 25% through cavitation (sonication) [17]. The effect of cavitation phenomena on solid materials is evident by observing the holes produced by the jet caused by the collapse of bubbles on an aluminium sheet in less than 1 min (Figure 4), these holes being obtained in various operational conditions, such as with or without degassing in a static or flowing solution [18].

In addition to fragmentation, a major change in the structure of the materials was also reported. Thus, in the case of terpene extraction from cumin, a change in selectivity was observed in the case of using cavitation phenomena at the same extraction yield. Conventional extraction empties the cells but leaves their structures intact, while the degradation of the cell walls occurs when ultrasound is applied [19]. This destruction of the cells may be the mechanism of the destruction of pathogens in the treatment of fruit with ultrasound.

The capillarity effects of the solvent in the structure of the material from which the extraction is performed are influenced by ultrasound, which leads to an increase in the depth and speed of penetration of the liquid into the channels and pores [20]. The ultrasound-assisted extraction of polyphenols from apple pomace led to improved extraction kinetics, with the major difference between the two extraction processes occurring in the first 10 min of extraction [21]. The difference was due to water absorption that favoured the diffusion of polyphenols from the jujube, which was experimentally demonstrated by measuring the water retention capacity, which was approximately 70% higher in pomace treated with ultrasound compared to maceration [16,22]. The degree of swelling of plant material increased from 5 to 10% by the use of ultrasound, leading to an increase in extraction, with the impact of ultrasound on the basic mechanisms of extraction including the desorption and diffusion of a solute from a plant structure [23].

Sonoporation, represented by the pores in cell membranes through the use of ultrasound for the transfer of nucleic acid material, is inevitably a phenomenon also involved in extraction processes. For sonoporation, ultrasound with high frequencies (over 500 kHz) is applied, but also the use of lower frequencies (20 kHz) with cell wall permeabilisation effects [24]. For extraction, sonoporation can be used to create pores in the cell membrane that are reversible or irreversible, leading to the release of cell contents into the extraction medium. Materials treated with ultrasound show a strongly impacted surface and perforations of the membrane, caused by cavitation, which leads to a higher extraction yield [25].

## 4. The Parameters That Influence Ultrasound-Assisted Extraction

Several parameters influence ultrasound-assisted extraction: physical factors (power, intensity and frequency of ultrasound), the geometry and size of the extraction reactors, the working environment (solvent, temperature, pressure, dissolved gases, additional mixing) and the material from which the extraction is performed.

**Figure 5:**
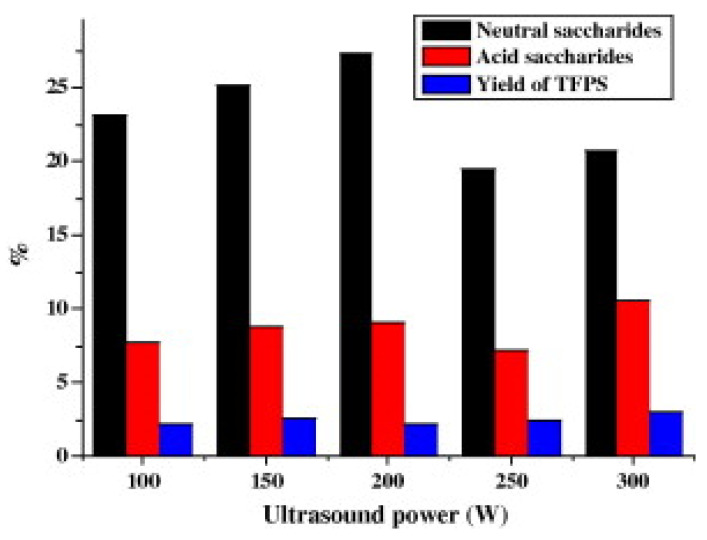
Effect of the ultrasound power on the composition and yield of tea flower polysaccharides (TFPS). Reproduced from [27] with permission.

The applied power is not transferred in a useful way to the environment, and the energy can be measured either by physical methods, using hydrophones, calorimeters or optical microscopes, or by chemical methods, by quantifying the radicals formed in the environment [16]. The induced effects on the plant material vary depending on the power of ultrasonication, but optimisation also aims to minimise costs (the minimum power for a result considered acceptable even if it is not the theoretical maximum) [26]. In addition, depending on the power applied during the extraction, the selectivity of the obtained species can be obtained. For example, the extraction yield of polysaccharides from tea flowers varied slightly with a change in ultrasonic power between 100 and 300 W, but the composition of the extract in neutral saccharides and acidic saccharides changed irregularly depending on the power of ultrasound [27]. It was observed that both the composition and bioactivity of tea flower polysaccharides varied for extraction using ultrasound powers set at 127.5, 300, 495, 637.5 and 750 W: the yield of tea flower polysaccharides changed only slightly with the increasing ultrasound power, but, for higher ultrasound power, the authors extracted different ratios of neutral and acid saccharides [27], as presented in Figure 5.

The ultrasound frequency influences the cavitation processes, so if the frequency increases above a certain value, the intensity of the cavitation in the liquid decreases. For very high frequencies, the compression–decompression cycles of the medium can be too short to lead to the formation and growth of cavity bubbles, because the duration of the decompression phase during which the cavity bubbles grow is inversely proportional to the ultrasonic frequency [28]. The most frequently used frequencies are between 20 and 100 kHz, a reduced impact being observed at 500 kHz compared to 20 kHz [29]. Moreover, the frequency has an influence on the selectivity of the extraction; for example, the phenolic substances from peanuts at an applied frequency of 25 kHz had a higher concentration of daidzein and genistein, and, at 80 kHz, the concentration of biocanin A and trans-resveratrol increased [30]. The frequency also influences the time required for extraction. For example, polyphenols from grape pomace were obtained faster at 40 kHz compared to 80 and 120 kHz [31].

The intensity of ultrasound (energy transmitted per time and surface) is dependent on the amplitude of the sonicator. The collapse of the bubble becomes stronger with the increase in the pressure amplitude and a minimum intensity value is required to pass the cavitation threshold. The sonication intensity has a strong impact on the extraction yield, and the amplitude depends on the viscosity of the extraction medium when working with high-viscosity liquids, such as oils [9]. Soybean oil extraction increased with the sonication intensity for all tested values: 16.4, 20.9, and 47.6 W/cm2, respectively [32].

The shape of the vessel (reactor) in which the extraction takes place is very important because ultrasound is reflected when it meets a solid surface. Moreover, the ultrasound source (bath or probe) is very important. In the case of ultrasonic baths, the best choice is a vessel with a bottom to minimise the reflection of the waves [33]. The thickness of the vessel is also important and must be minimal to reduce the attenuation of ultrasound in transit. In the case of using a probe immersed in the extraction medium, several parameters are important, such as the position of the emitter in relation to the transducer, the shape and size of the probe or the distance between the ultrasonic probe and the vessel wall [9].

The choice of solvent is determined by the good solubility of the target substance, which is essential for any type of extraction, but also by physical parameters such as viscosity, surface tension and vapour pressure, which influence the cavity. Viscosity and high surface tension imply an increase in molecular interactions in the solvent, which leads to cavitation initiation at a higher energy threshold, which creates sufficient negative pressure during the decompression cycle to overcome the cohesive forces between the molecules that compose the liquid [28]. Solvents with low vapour pressure are preferred because the collapse of the cavity bubble is more intense compared to solvents with high vapour pressure [34], but this factor can be adjusted by increasing the working temperature.

The temperature is chosen in close correlation with the solvent as it influences its properties. An increase in temperature leads to a decrease in both viscosity and surface tension and induces an increase in vapour pressure, as mentioned in the previous paragraph. Apart from cavitation effects, the temperature influences extraction processes in general, but, in the case of sonication, the effect is cumulative. On the other hand, ultrasonication allows the extraction temperature to drop, which is beneficial in the case of compounds that can be degraded or hydrolysed. An example is turmeric, a natural dye that is widely used in the food and textile industries. Ultrasound-assisted extraction from the rhizomes of turmeric plants was performed using a double-walled vessel to maintain the temperature at 25 °C and avoid the temperature increase caused by exposure to ultrasound, obtaining an extraction yield approximately three-times higher than the traditional method. At the same time, it was found that the extraction of curcuminoids increased when increasing the extraction time, but when the extraction time was longer than 15 min, the extraction yield decreased with increasing time, indicating that the induced cavity can degrade the compounds when they are exposed to a longer period of time, and this result demonstrates the need to optimise the parameters [35].

The presence of dissolved gases in the extraction liquid is important in regard to the cavitation phenomena because the formation of gas bubbles is facilitated precisely by the presence of gases, which act as nuclei for a new cavity bubble [28]. In the absence of gases, the cavitation process is based on the evaporation of the solvent, which inevitably leads to a reduced process [36]. The sonication process has the effect of removing the gases dissolved in the environment over time, and, as a result, a gas can be bubbled for refreshment, but this operation is not usually applied and the control of the dissolved gases is not implemented [16].

## 5. Commercially Available Ultrasound Sources

**Figure 6:**
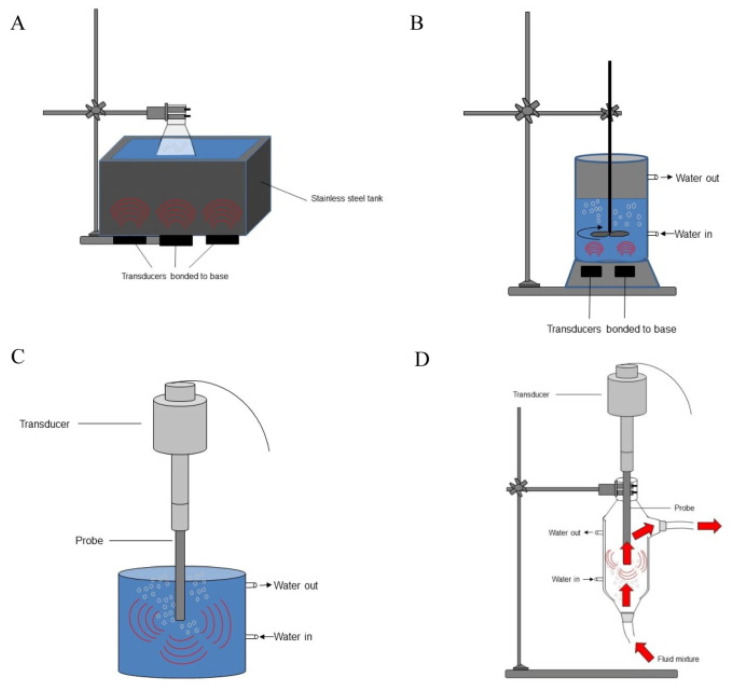
Examples of ultrasonic extraction systems (**A**) bath, (**B**) reactor with stirring, (**C**) probe, (**D**) flow system with ultrasound probe. Reproduced from [16] with permission.

In the case of extractions performed in the laboratory, two types of ultrasound sources are used: baths and probes (Figure 6). Ultrasonic baths have the advantages that they can simultaneously contain several extraction vessels, the probes are inserted directly into the extraction medium and the power is not variable depending on the material and thickness of the extraction vessel, while the probes are more flexible in terms of vessels and volumes used.

There is a wide variety of ultrasonic baths from numerous manufacturers, a representative selection being presented in Table 1. These devices are developed for other applications (industrial cleaning) but can also be used directly for chemical extraction. It can be observed that the sonication frequency is generally 40 kHz, but there are also manufacturers that deliver devices at 25–28 or 80 kHz, and even a simultaneous combination of several sources with different frequencies. The industrial devices have a wide range of technical characteristics, which allows the choice or testing of different conditions for the extraction of fruit compounds and the optimisation of work parameters. An interesting option is the possibility to regulate the heating of the working liquid with the help of additional resistance, taking into account that ultrasonication is a process that normally leads to the heating of materials through energy dissipation.

Regarding ultrasonic probes, their construction is different from that of baths, as the source is fixed at the top of a rod instead of at the bottom of the bath. For this reason, the transducer is smaller and the distribution of ultrasound in the extraction medium is different, the origin of the ultrasound being small compared to baths, which ensure more homogeneous ultrasound in the medium. As for the sizes of the ultrasonic probes, they are very varied, from hand-held portable devices for small volumes to medium-sized devices for laboratory extractions or large ones adapted to industrial extractors. Moreover, the probes usually used in batch systems can be adapted for flow systems by using adapted cells. As far as ultrasound probes are concerned, there are many commercially available devices that can be used for the extraction of active products. Table 2 shows some examples from different manufacturers, and it can be seen that the range of frequencies and powers is similar to that of ultrasound baths; it should be mentioned that the initiation of ultrasound is rapid, and the comparisons and the effects on the material are not identical.

## 6. Examples of Extractors That Include Ultrasound

**Figure 7:**
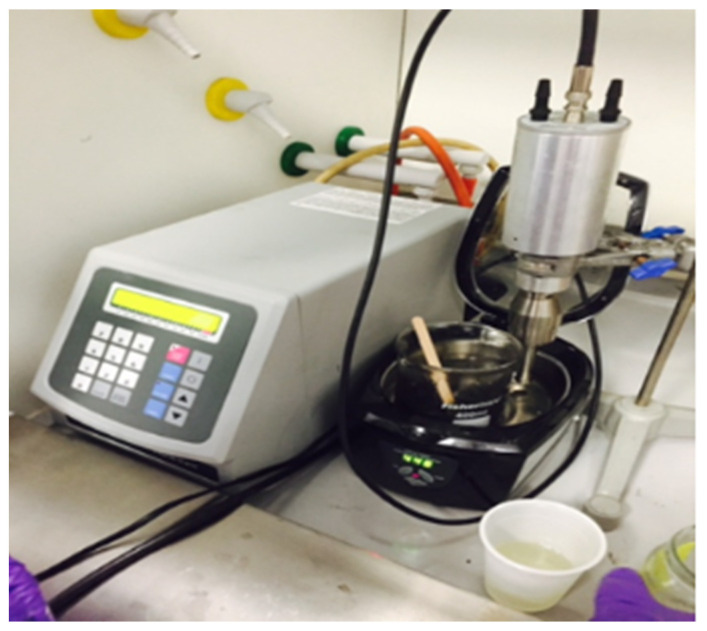
A combination of bath sonication, tip sonication and manual stirring for the complex treatment of the media. Reprinted from [38] with permission.

There is a wide variety of extractors that also include the ultrasonication of samples. Depending on the stage of technological development, sample size and application, either batch or flow devices can be used (Figure 6). Thus, the simplest system consists only of the extraction vessel, in which the sample is mixed, and the solvent, in which an ultrasonic probe is inserted. Obviously, this system is useful for initial studies when it is desired to test different combinations of raw materials, solvents and ultrasonication conditions. The next step consists of creating a system with a flow that ensures control of the contact between the solvent and the raw material. Moreover, thermostating is important because ultrasonication transfers a relatively large amount of energy in a small volume of extraction, which leads to an increase in temperature. This increase in temperature can non-specifically help the extraction, as in any classic maceration, but it must be controlled in the case of unstable compounds. Regarding applications on an industrial scale, they are carried out in complex installations that ensure the precise control of the process and integrate several processes in a single system, besides ultrasonication and filtration or recirculation of the solvent, in order to obtain the maximum economic effectiveness (Figure 6). Interestingly, different ultrasound sources can be combined that do not necessarily have the same working frequency [37]—for example, combining a probe with a bath in a common sample processing system [38], as presented in Figure 7.

**Figure 8:**
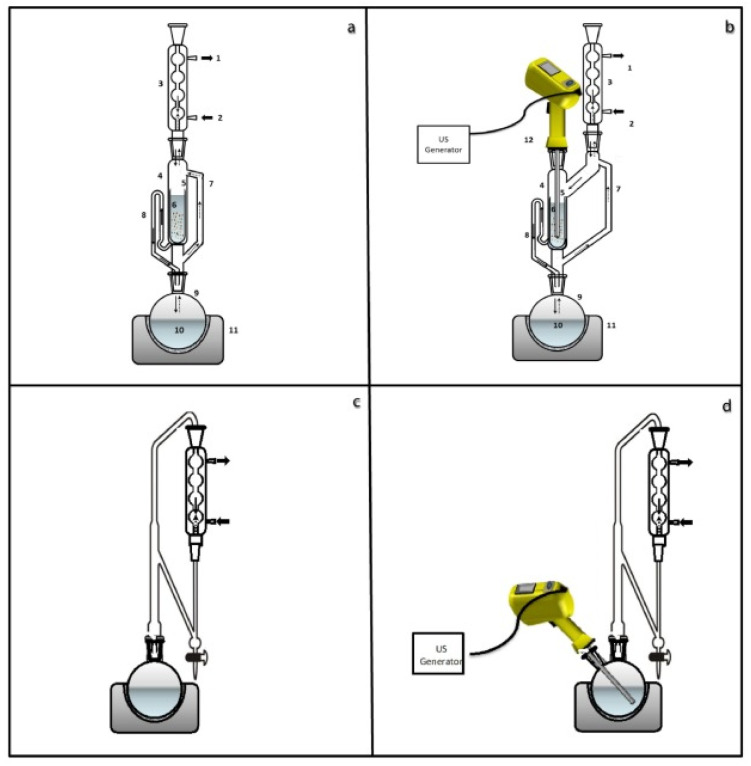
Conventional and ultrasound combined extraction techniques: (**a**) conventional Soxhlet, (**b**) sono–Soxhlet, (**c**) conventional Clevenger, (**d**) sono–Clevenger. Reproduced from [16] with permission.

An extraction method with increased effectiveness is the Soxhlet system, which is the reference technique for a wide range of compounds in solid–liquid extraction. This method involves the repeated percolation of a fresh solvent through a solid matrix and presents a number of advantages over simple solvent extraction (maceration), such as smaller amounts of solvent used, reduced extraction times and better reproducibility. The solid phase is repeatedly linked to a fresh volume of solvent generated from the condensed vapour until it reaches a certain level, which triggers the return of the solvent to the tank. Despite the recognised efficiency of this process, Soxhlet extraction has certain shortcomings in terms of the time required for complete extraction and the possible decomposition of some analytes recovered in the boiling solvent tank. Improvements to this classic technique to reduce the extraction time, the amount of solvent used and/or the energy consumption can be obtained by integrating ultrasound into the process. These systems were developed either by using an ultrasonic probe submerged in a water bath circulating around the Soxhlet system, or by inserting an ultrasonic probe directly into the extraction chamber. This innovative use of ultrasound in the Soxhlet process leads to a reduced extraction time without degrading the extracts, and the application of ultrasound directly in the extraction reactor optimises the process by avoiding indirect transfer from the water bath through the glassware and then to the solvent surrounding the cellulose cartridge in the Soxhlet chamber [39]. In a similar manner, the conventional Clevenger extraction method can be combined in an improved sono–Clevenger configuration [16], as presented in Figure 8.

## 7. Effects of Cavitation Phenomena on Dairy Products

Various effects on dairy products are induced depending on the high or low intensity of the ultrasound waves. Low-intensity waves are used to control the quality of dairy products, detecting physical–chemical variations during storage or processing that change the transmission of sound through the environment. High-intensity ultrasound induces physical, mechanical and chemical changes through cavitation phenomena and is used for processing applications, such as creaming, pasteurisation, homogenisation, reducing the allergenicity of milk proteins, reducing the viscosity or extraction [40].

Ultrasound applied to milk has the effect of inducing the production and release of numerous volatile compounds, such as aldehydes, ketones, esters, alcohols and hydrocarbons, which are produced by lipid oxidation or protein degradation. Volatile compounds are detected both in pure fats and in mixtures of casein and fat systems. The amount of milk fat has a minor influence on the production of volatile substances, but the sonication time is important. Thus, for sonication times less than 5 min, the milk has a profile of volatile substances similar to the initial one (untreated), but for prolonged sonication, the production of volatile compounds increases considerably. For this reason, the method must be applied for short periods in order not to change the taste [41]. High-intensity, low-frequency ultrasound treatment also has an influence on milk fat. Prolonged treatment led to predominant increases in the ester groups corresponding to lipids or complexes of lipids and proteins and slightly influenced the secondary structural changes of whey proteins or caseins. The acoustic cavity also induced structural changes in proteins due to denaturation/aggregation, and the disruption of milk fat globules/fat clusters led to new protein–protein and protein–lipid interactions [42].

**Figure 9:**
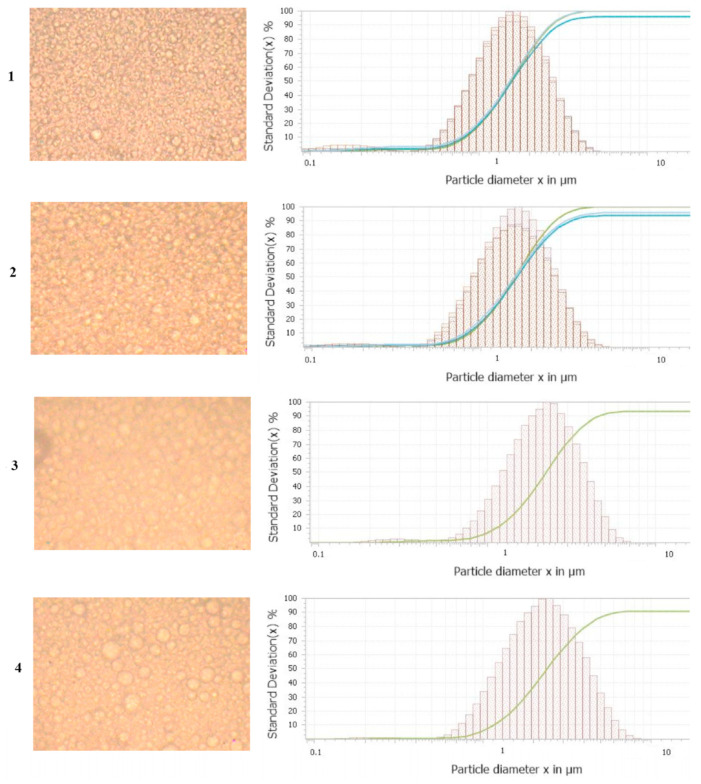
Micrographs of camel milk creams as affected by thermosonication treatment (power, time and temperature of sonication): (**1**) 60 W, 6 min and 65 °C; (**2**) 90 W, 6 min and 45 °C; (**3**) 30 W, 1 min and 45 °C; (**4**) 30 W, 3.5 min and 25 °C. Reproduced from [43] with permission.

The working temperature also has an important influence on the composition of the milk. For example, with an increase in sonication power, time and temperature, the average size of the fat globules decreased (Figure 9). On the other hand, when increasing the sonication power and time, the increased stability over time of the emulsion was obtained, this being inversely proportional to the increase in temperature [43].

Regarding the effect of sonicating raw milk at different temperatures for the purpose of microbial inactivation (pasteurisation at a lower temperature), a reduction in the total number of bacteria was demonstrated, which was primarily attributed to the increase in temperature during the treatment, without denaturing the whey protein or the formation of gels [44]. An appropriate choice of sonication power and its combination with a thermal treatment applied at a relatively low temperature (57 °C, a borderline temperature that does not cause unwanted physico-chemical effects) can obtain a combined effect of microbial inactivation accompanied by a decrease in alkaline phosphatase activity and a significant reduction in the size of fat globules, which leads to good milk storage stability [45]. Interestingly, the sonication of milk can also be used to increase the activity of microorganisms and not simply to inactivate them. Fermentation is one of the most important stages in the processing of dairy products, but it is also one of the most time- and resource-consuming stages during production. Ultrasound can improve the fermentation process of lactic acid bacteria by changing their metabolic activity, reducing, at the same time, the fermentation time and improving the quality characteristics of the fermented dairy product. The difference between the activation and inactivation of microorganisms by sonication is obtained by changing the working parameters: the power and frequency of ultrasound, sonication time, type of microorganism, pH and temperature [46].

In the production of yogurt, sonication is used for homogenisation and emulsification by reducing the size of milk fat particles, increasing the viscosity and water retention capacity by reducing liquid loss, improving the resistance and firmness of the gel by increasing the coagulation properties of whey proteins, reducing the fermentation time by improving lactose hydrolysis and stimulating probiotic bacteria. In the production of ice cream, sonication applied during freezing has the effect of reducing the size of the ice crystals, decreasing the freezing time and preventing binding to the packaging [47].

The instant rehydration of protein-rich milk powder is favoured by ultrasonication in combination with vigorous mixing, commonly used in industrial facilities. Particle size distribution using only conventional intensive mixing does not lead to the complete rehydration of milk powders, but its combination with ultrasonication leads to complete rehydration. The apparent viscosity decreased significantly after ultrasonication compared to dispersions subjected to conventional mixing, and the reconstituted milk had increased sedimentation stability compared to the mixing-only samples. Ultrasonication has the potential to achieve the complete rehydration of powders in significantly less time than conventional rehydration processes used by the dairy industry [48].

## 8. Cavity-Based Extraction from Dairy Products

Numerous extraction methods that also use the cavitation phenomenon have been developed for different applications. Cavitation is coupled with classic extraction methods such as filtration or solid-phase extraction to decrease the working time or improve yields. Classical extraction procedures, such as liquid–liquid extraction or Soxhlet extraction, are techniques that require a large amount of time and large quantities of organic solvents. As an alternative to these classical methods, ultrasound-assisted extraction is an ecological procedure for sample preparation and analyte extraction from different matrices. The cavitation process produced by ultrasound radiation considerably reduces the extraction time required in traditional solid-phase extraction procedures [49]. The effective sample/extractant contact provided by sonication leads to the good recovery of the analyte in the extraction process. Ultrasound in membrane filtration does not influence the intrinsic permeability of the membranes, but only prevents the blocking of the membrane. Low frequencies, higher power intensities, intermittent sonication, lower viscosities, lower temperatures and higher pressures are the most important factors to improve the membrane blocking prevention and flux [50]. The integration of an ultrasound emitter in a unit with a skim milk ultrafiltration membrane allowed a significant increase in the amount of permeate, even for intermittent operation in cycles of sonication [51]. Two sources of ultrasonic radiation, ultrasonic baths and ultrasonic probes, are commercially available to provide different frequencies and powers in ultrasound. Ultrasonic probes are applied mostly in industrial applications, while ultrasonic baths are more widely adopted in laboratory research, but the lack of uniformity in the distribution of ultrasonic energy and the decrease in power over time can often decrease the repeatability and reproducibility of the extraction efficiency [52].

The determination of numerous antibiotic residues (17 quinolones and 14 β-lactams) from raw cow’s milk samples was carried out following the extraction of the analytes assisted by ultrasound, followed by a cleaning step using a dispersive solid-phase extraction sorbent. The selected extraction solvent was a mixture of acetonitrile/methanol/pH 6 buffer solution that was added to freeze-dried milk samples, and the mixtures were sonicated for 20 min at 70% amplitude and analysed by UHPLC-ESI(+) -MS/MS. The extraction method has the advantage of eliminating the difficulties of extracting highly water-soluble compounds, such as these classes of antibiotics, from the aqueous matrix [53]. Different solid sorbents have been used in ultrasound-assisted sample pretreatment—for example, organometallic structures (MOF) synthesised from copper and 1,3,5-tricarboxylic acid for the selective, sensitive and rapid determination of mefenamic acid [54]. Alternatively, ampicillin has been analysed using MOFs composed of copper and terephthalic acid that also contain Fe_2_O_3_ nanoparticles, allowing it to be magnetically separated after the ultrasound-assisted extraction process [55]. Moreover, molecularly imprinted polymers (MIP) were used to extract samples such as nicotinamide (vitamin B3) from milk, requiring a small sample volume and a sonication time of only 5 min [56].

Various deeply hydrophobic eutectic solvents with quaternary ammonium salts and salicylate esters as hydrogen bond donors have been used as extraction solvents for ultrasound-assisted dispersive liquid–liquid microextraction. A solvent of tetrabutylammonium chloride and methyl salicylate with low viscosity allowed the extraction and analysis of phenolic endocrine disruptors from milk samples by chromatographic means. Several key parameters affecting the extraction efficiency were investigated, including the solvent type and volume, sonication time, sample solution pH and salt addition. Ultrasound promotes the dispersion of eutectic solvents in the aqueous solution and accelerates the mass transfer of analytes from the sample phase to the solvent phase. The extraction efficiency, depending on the sonication time, increased gradually from 0 to 6 min and then remained unchanged, which indicated both the importance of sonication and the fact that only a short working time was required [57]. A liquid–liquid microextraction procedure using sugar-based eutectic solvents assisted by ultrasound for the extraction of aflatoxin M1 from milk samples was developed. Different eutectic solvents were prepared by mixing and heating choline chloride or betaine chloride with xylose, fructose, sucrose or maltose to obtain a homogeneous and viscous liquid. The extraction procedure consisted of three steps: (i) pyrogallol and sugar-based eutectic solutions were added to the sample solution at pH 3.8; (ii) ultrasonication of the mixture was performed for 8 min to mix the solvent with the sample and extract the analytes; and (iii) centrifugation was conducted to achieve separation from the mixture [58]. Such deeply hydrophobic eutectic solvents have been developed for the analysis of other analytes, such as non-steroidal anti-inflammatory drugs by chromatography after ultrasound-assisted dispersive liquid–liquid microextraction, using guanidinium chloride and thymol to obtain the solvents [59].

The analysis of heavy metals usually involves the mineralisation of the sample and requires concentrated acids and high temperatures for matrix digestion, which is not in accordance with the principles of green chemistry. Microwave-assisted extraction is another sample preparation strategy that has proven to be very effective for metal determination, but this methodology is also expensive, consumes more energy and is not as safe and fast as ultrasound-assisted procedures. Ultrasound allows the treatment of samples simply and quickly, without external heat and using dilute acid. Thus, the reference method consists of adding over 0.5 g of the sample to 15 mL of concentrated nitric acid and 2 mL of 30% hydrogen peroxide and heating it on a hot plate at 100 °C for 15 min. By comparison, the ultrasound-assisted method using a probe requires 25.0 ml of 15% nitric acid for the same sample mass and a sonication time of 5 min. Instead of the probe, an ultrasound bath can be used, both alternatives producing results similar to the classical method [60]. The rapid sequential determination of six elements (Zn, Fe, Mg, Ca, Na and K) using flame atomic absorption spectrometry was performed after pretreatment of the samples using nitric acid and sonication for 10 min at 80 °C, the results being confirmed by the microwave digestion of samples, which confirmed the usefulness and viability of using ultrasound as an alternative method [61].

Apart from analytical chemistry, ultrasound-based extractions are also used to obtain biologically active compounds such as enzymes. Lactoperoxidase is found in milk whey in a concentration of around 30 mg/L, constituting approximately 0.5% of whey proteins. Conventional extraction techniques have several stages, are time-consuming and lead to a loss of activity. Aqueous extraction in two phases consists of dividing the biomolecule between the two aqueous phases of a system formed by mixing either two polymers or a polymer and a salt. Lactoperoxidase was separated from whey by combining two different processes: aqueous extraction in two phases and ultrafiltration. Ultrasound has been used to improve the performance of the ultrafiltration process and avoid a decrease in the permeate flow by avoiding the blockage of the membrane [62].

## 9. Effect of Cavitation Phenomena on Fruit

Fruit, along with vegetables, are widely consumed due to their nutrients and bioactive compounds. Due to the fact that fruit are alive, they show respiratory and metabolic activities, which easily cause water loss, microbial infection and quality deterioration during transport and storage [63]. Since fruit are not always consumed directly, a field of wide interest is the extraction of substances of interest from fruit and secondary materials through fast procedures that avoid the denaturation of biocompounds. For this purpose, innovative technologies are being investigated that bring different advantages that can be combined: for example, the cavity allows both the sterilisation of the fruit and improved extraction of the active principles. The cavitation phenomena lead to high shear forces, and the implosion of the cavitation bubbles leads to microjets that generate several effects, such as surface exfoliation, erosion and particle decomposition, the combination of which contributes to the final effect [16]. In addition, fruit have a short preservation time, with high losses through degradation, and methods are needed to extend their preservation times. Moreover, fruit are easily contaminated by microorganisms such as *Escherichia coli*, *Salmonella* or *Listeria monocytogenes*, which can cause outbreaks of foodborne diseases [64]. To reduce the risk due to pathogens, fruit are washed with disinfectants such as chlorine [65], but the use of chlorine can induce the formation of halogenated organic compounds that endanger the health of consumers [66]. Consequently, new technologies are needed to improve the safety of fruit, as they can simultaneously be used for other purposes in the same treatment/extraction process.

**Figure 10:**
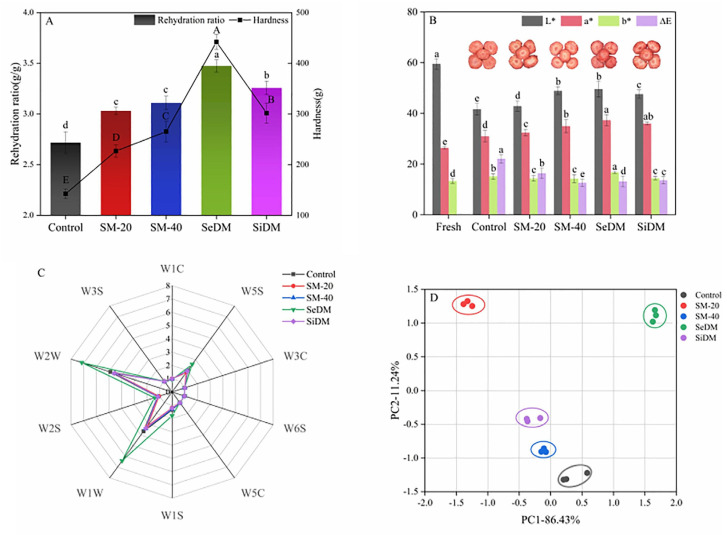
Effect of pretreatment on the quality of strawberry subjected to vacuum freeze-drying. (**A**) Effect of pretreatment on rehydration ratio (bar graph) and hardness (line graph) of strawberry after vacuum freeze-drying; (**B**) effect of pretreatment on the colour of strawberry after vacuum freeze-drying; (**C**) radar chart of the effect of pretreatment on flavour substances of strawberry after vacuum freeze-drying; (**D**) effect of pretreatment on main components of flavour substances of strawberry after vacuum freeze-drying. The frequency modes are single-frequency mode under 20, 40 kHz (SM-20, SM-40) and dual-frequency mode under 20/40 kHz including sequential mode (SeDM) and simultaneous mode (SiDM). The a–e for each graph/sample represents the groups that are statistically different. Reproduced from [69] with permission.

Different innovative techniques have been tested and proposed for the treatment, processing, extraction and preservation of fruit, such as high pressure, a pulsed electric field, ultraviolet light, gamma irradiation and ultrasound [67]. Among these technologies, ultrasound has advantages such as a low cost, respect for the environment, a lack of toxicity and not requiring additives [68]. Ultrasound has been tested for different purposes in fruit treatment, such as vacuum freeze-drying [69], starch degradation [70], enzymatic hydrolysis [22] and freezing and thawing [71]. Due to the complex effects that cavitation phenomena have on the sample, it is not possible to clearly separate the pretreatment (for example, crushing or homogenisation under the effect of ultrasound) from the actual extraction process. The ultrasound pretreatment before vacuum freeze-drying influences various aspects of fruit, such as the rehydration ratio, hardness, colour and flavour, as a function of the frequency and operation mode. Moreover, the drying time of strawberry slices was significantly reduced by ultrasound, with the dual-frequency mode being more effective than single-frequency ultrasound. In addition, the quality characteristics of dried strawberry products pretreated by dual-frequency ultrasound were significantly better than those of control and single-frequency pretreated fruit (Figure 10) [69].

## 10. Cavity-Based Extraction from Fruit

The peel and pomace obtained after the fresh juice was squeezed were used to obtain extracts containing limonoids and various polyphenols, including naringenin or hesperetin, and fatty acids such as palmitic, myristic and linoleic acids. The ultrasound-assisted method (30 °C, 200 W, 40 kHz for 45 min) used a hydroalcoholic solvent and allowed the researchers to obtain some compounds with antioxidant activity that reduced the cell viability of different tumour cell lines, which allowed the recovery of waste [72].

Carotenes and their esters were extracted using ultrasound from papaya pulp and peel, using soybean oil and sunflower oil as alternative green solvents. The maximum total content of carotenoids was obtained from papaya pulp extracts using soybean oil or sunflower oil 10 min, with an amplitude of 60%. On the other hand, the maximum content of carotenoids from papaya peel was obtained using soybean oil when applying a 20% amplitude for 77 min/20% ethanol. This demonstrates the importance of optimising the process depending on the type of raw material, even for the same fruit, since the properties of the tissues and cells are different and thus the mechanical effects of the ultrasound on them are also different [73].

The ultrasound-assisted extraction (20 kHz probe, 70 W) of anthocyanins from blackcurrant samples had similar yields to enzyme-assisted extraction for the purpose of breaking down cell walls; in addition, it presented some advantages, e.g., the work time was reduced by half and it did not require expensive bioreagents that could contaminate the products. Both methods showed high precision and repeatability, with RSDs below 5%, which makes them suitable for quality control analysis [74].

The efficient extraction of pigment from green chestnut peel was also achieved using an ultrasound-assisted method in combination with ionic liquids. The results indicated that the properties of anions and cations had a significant impact on extraction, and 1-butyl-3-methylimidazolium acetate had the best extraction effect. Compared to the conventional extraction with reflux with organic solvents (sodium hydroxide, ethanol), the optimised method improved the yield and allowed the researchers to reduce the working time. The power of the ultrasonic bath did not have an important influence on the extraction yield in the range of 100–700 W, but the extraction time was important [75].

Ultrasonic extraction was combined with microwave and natural eutectic solvents (choline chloride, glucose, water) for the efficient extraction of pectin from dragon fruit peels. The combination of ultrasound with microwave allowed the extraction of pectin with a higher yield and higher quality. The sequential use of microwave and ultrasound offers better efficiency than individual techniques [76]. Moreover, the extraction of mango kernel oil was improved by combining microwave-assisted extraction with ultrasound, reaching oil recovery of 97% [77].

Ultrasound-assisted three-phase partition extraction improved imperatorin extraction from Bengal quince (*Aegle marmelos*) fruit. Extraction by partition in three phases involves the addition of salt (ammonium sulphate) to the aqueous mixture of the crude extract with the raw material, followed by the addition of an organic solvent (t-butanol). Water and t-butanol are completely miscible with each other, but a sufficient amount of ammonium sulphate separates the extraction mixture into three phases: the upper layer is an organic phase, which mostly contains hydrophobic components such as pigments and lipids; the middle phase is the phase rich in proteins or enzymes, while polar components, such as carbohydrates, are accumulated in the lower aqueous phase. The optimised parameters were as follows: sonication time 30 min, ammonium sulphate mass 30% (*w*/*v*), biomass to solvent ratio 1:40 (*w*/*v*), biomass suspension to t-butanol ratio 1:1 (*v*/*v*), 120 W ultrasonic power and 60% duty cycle (6 s to 4 s off). The maximum extraction yield of imperatorin was 5.44 mg/g, which was much higher than that obtained with traditional techniques such as three-phase partitioning (TPP), Soxhlet and batch extraction [78].

**Figure 11:**
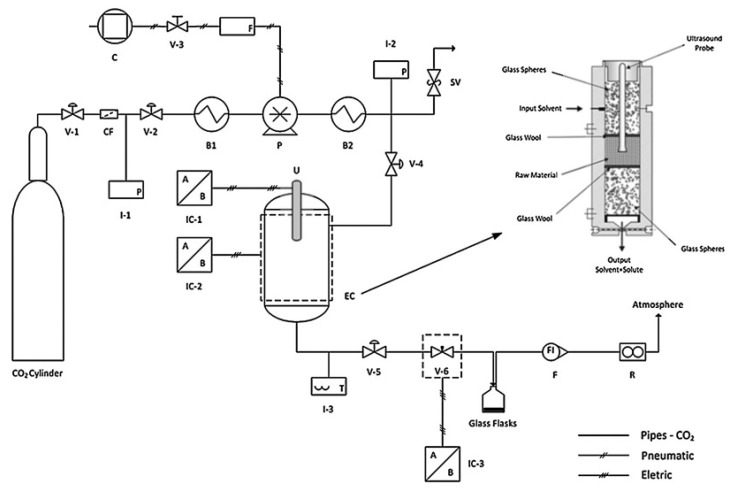
Diagram of the ultrasound-assisted supercritical extraction unit used for high-yield oil extraction from passionfruit seeds. The symbols are sufficient: valves, controllers, etc. Reproduced from [79] with permission.

While most of the extraction processes reported use relatively simple equipment, representative of research laboratories in the early stage of technological readiness, for transfer to industrial applications, more complex installations must be developed, such as the combination of ultrasound with supercritical CO_2_ for oil extraction from passionfruit seeds. Such equipment is obtained using complex components, such as control valves, safety valves, compressors, filters, cooling/heating baths, pumps, pressure/temperature indicators, controllers of ultrasound power, flow meters and extraction columns (Figure 11). In the mentioned study, the application of ultrasound increased the yield by up to 29% due to the fact that ultrasound released the extractable material from inside the particles at moderate pressure [79].

## 11. Conclusions

In conclusion, cavitation phenomena have wide-ranging applications; besides mechanic usage (material cleaning/cutting) or green chemical synthesis (activation), they are also useful for food treatment. Cavitation allows the improvement of the extraction of various substances of interest from fruit and dairy products, being able to improve the extraction yield but also reduce the denaturation of labile substances, the use of chemical compounds (solvents, enzymes, surfactants), the temperature and the working time. Interestingly, cavitation is also a highly flexible technique with multiple applications in food production processes (mixing, pathogen reduction, particle size control, etc.) to achieve the desired final products. The method is applied both in the laboratory (studies and analyses) and on the industrial scale, but its up-scaling is not straightforward due to the physical phenomena that occur (e.g., excessively high local power if a large power source is directly fitted for a larger volume). Further developments aim for the greater integration of the cavitation phenomena into the technological workflow, since sonication is not an aim but a tool that contributes to the overall process.

## Figures and Tables

**Table 1:** The main characteristics of commercially available ultrasonic baths.

Manufacturer	Power	Frequency	Capacity	Observations
Jeken	40 kHz	30 W	450 mL	Developed for home cleaning
	42 kHz	120 W	2.5 L	Product intended for dental offices
	40 kHz	720W	45 L	Industrial applications
Ultratecno	28 or 40 kHz	1–36 kW	113–13,258 L	Industrial cleaners
Elmasonic	37 kHz	120–960	3–90 L	Different sonication modes: pulse, continuous, variable frequency
Zenith Ultrasonics	25, 40, 80 kHz			Combining several frequencies in the same bath for multiple effects

**Table 2:** The main characteristics of some ultrasonic probes.

Producer	Frequency	Power	Capacity	Observations
Hielscher	26 kHz	200 W	0.5–1.5 mL	Very small volumes
	30 kHz	100 W	0.01–500 mL	Laboratory variable volumes
	24 kHz	400 W	5–2000 mL	Large volume laboratory
	18 kHz	16 kW	0.2–50 mc/h	Industrial applications
Silabtec	35 kHz	120 W	0.2–50 mL	Axial transmission
	20 kHz	500 W	20–500 mL	Axial transmission
	20 kHz	750 W	100–100 mL	Radial transmission in the test
	20 kHz	750 W		Adapted for tubes (flow)

## Data Availability

Not applicable.
